# Relationship between Tumor Cell Invasiveness and Polyploidization

**DOI:** 10.1371/journal.pone.0053364

**Published:** 2012-12-31

**Authors:** Javier Mercapide, Fabio Anzanello, Germana Rappa, Aurelio Lorico

**Affiliations:** Cancer Research Center, Roseman University of Health Sciences, Las Vegas, Nevada, United States of America; University of Michigan School of Medicine, United States of America

## Abstract

A number of studies have shown that tumor cells fuse with other tumor and non-tumor cells. In the present study on tumor cell lines derived from glioblastoma, breast cancer, and melanoma, we estimated the frequency of fusion between tumor cells by establishing the fraction of cells with whole tumor-genome duplication in each cell line. Together with this, the capacity of the tumor cell lines to spread through a basement membrane scaffold was assessed, in order to test the hypothesis that pericellular proteolysis by enzymatic release in the spaces of intercellular contact could account for differences in the fusogenicity of tumor cells. The difference in invasiveness between the cell lines accounted for their specific amount of cells with tumor-genome duplication, which, depending on the cell line analyzed, ranged from 2% to 25% of the total cells. These results support the hypothesis that cell-to-cell invasion eliciting membrane fusion causes polyploidization in tumor cells.

## Introduction

The implication of aneuploidy in the initiation of the carcinogenic process has been argued in recent years [1]. According to the aneuploidy hypothesis, tumorigenicity would arise in aneuploid cells that surpass a threshold of deregulation and reacquire some degree of mitotic stability [2], [3]. Given that cell fusion produces polyploidization, which is associated with chromosome mis-segregation during mitosis and generation of aneuploidy [4], discerning the degree of implication of cell fusion in the processes of transformation and tumor progression appears compelling. Experimental results dating back to the 1960s have established that tumor cells have the capacity to fuse with different types of tumor and non-tumor cells [5]–[7], leading to the hypothesis that tumor progression results from the mixture in fused cells of characteristics of two dissimilar cells. More recent work has supported this hypothesis, showing, in different tumor contexts, that cell fusion acts as a mechanism of genetic and epigenetic reprogramming [8]–[12]. Nonetheless, the significance of cell fusion in tumors is still elusive, owing to the fact that it remains unclear whether it is a frequent or a rather rare occurrence.

Pericellular proteolysis catalyzed by proteases secreted by cancer cells can form interstices in the tissues and facilitate cell movement and spread [13]. Hence, the release of proteolytic enzymes by a tumor cell at the point of contact with another cell, together with invasive movement, could determine fusion of their plasma membranes, thus influencing tumor cell fusogenicity. Here, the application of a method previously employed on glioma cells to detect cells with whole tumor-genome duplication [14] has been extended to cell lines derived from melanoma and breast tumors. We report that the levels of whole tumor-genome duplication are directly related to the ability of the cells to enzymatically decompose and break through a matrix layer. This suggests that extracellular lysis favoring the fusion of neighbouring cells plays a role in mediating genome duplication in cancer cells.

## Materials and Methods

### Cells, Tissue Culture, and Reagents

The human FEMX-I melanoma and MA11 breast carcinoma cell lines were derived from metastatic foci to lymph nodes and bone marrow, respectively [15], [16]. U87MG glioma and MDA-MB-231 breast carcinoma cell lines, and primary human fibroblasts, were purchased from the American Type Culture Collection. Cultures were kept in a humidified incubator at 37°C, in an atmosphere of 5% CO_2_, in either minimum-essential medium (U87MG and fibroblasts), RPMI 1640 (MA11 and FEMX-I), or Dulbecco’s modified Eagle medium (DMEM) (MDA-MB-231), supplemented with 10% fetal bovine serum (FBS) (Atlanta Biologicals), 2 mM L-glutamine (Hyclone), and 50 U/ml penicillin plus 50 µg/ml streptomycin (Lonza). 0.05% trypsin/0.5 mM ethylenediaminetetraacetic acid in Hank’s balanced salt solution and all the tissue culture media were from Mediatech Inc. All cultures were mycoplasma-free, as determined by PCR (Sigma) and DNA staining tests; changes in the original morphological characteristics of the cell lines were not observed. The stocks of the cell lines were stored in aliquots in liquid nitrogen, and extensive passaging in culture was avoided.

CD44 antibody, isotype control, and BB-2516 compound were procured from BD Biosciences, Southern Biotech, and Santa Cruz, respectively.

### Determination of the Cell Content of DNA

The quantification of the cell content of DNA was performed by measuring the intensity of the fluorescence emitted by individual cells after incorporation of propidium iodide into the DNA. To this aim, cells lifted by trypsinization were shaken repeatedly to get a single cell suspension and passed through a 70-µm cell strainer. After adding tissue culture medium with FBS at 10% for blocking trypsin, 20,000 cells were centrifuged at 200 g and resuspended in 0.5 ml of phosphate-buffered saline (PBS). Then, with a fine-tipped pipette, the cell suspension was aspired and ejected repeatedly to prevent aggregation. A 10-fold larger volume of PBS was then added, and the cells recentrifuged. The pellet was resuspended in 200 µl of PBS, and the cells added drop by drop to 10 ml under agitation of ice-cold 70% ethanol. Cells were kept at 4°C in the fixative solution for at least 1 h and then centrifuged at 430 g, resuspended in PBS containing bovine serum albumin at 1%, recentrifuged, and finally resuspended in 200 µl of Guava cell-cycle reagent (Millipore). The intensity of fluorescence in the cells by the DNA-intercalated propidium iodide was measured in a Guava EasyCyte™-Plus System using Cytosoft-5.3 data-acquisition and analysis software (Guava Technologies).

### Invasion Assays

Tumor cell invasiveness was assessed using BioCoat cell-culture transwell chambers with 8-µm-pore polyethylene terephthalate membranes coated with growth-factor-reduced matrigel (BD Biosciences). The matrigel layers were rehydrated with serum-free DMEM for 1–2 h. Cells were trypsinized and, after adding tissue culture medium containing FBS to the cell suspensions to block trypsin, centrifuged at 200 g, washed with serum-free DMEM, recentrifuged, and resuspended in serum-free DMEM; cells were counted in hemocytometer and the cell suspensions diluted with serum-free DMEM to contain 50,000 cells/ml. After removing the rehydration medium, the membrane inserts were dipped in DMEM with FBS at 2% in the assay chamber wells, and then 25,000 cells were seeded in each insert. The transwell plates were kept in CO_2_ incubator at 37°C for 18 h, and, at the end of the incubation, the cells remaining on the upper part of the membranes were scrubbed off with wet cotton swabs. The cells on the underside of the membranes were fixed with 4% p-formaldehyde for 10 min, and labeled with 1 µg/ml 4′,6-diamidino-2-phenylindole (Sigma) in PBS. Using a fluorescence microscope, the matrigel invading cells were counted in the central part of each membrane in 10 randomly selected areas 0.7-mm wide.

A paired transwell with 8-µm-pore non-coated membrane was included as control of chemotactic cell migration in each matrigel invasion test. Matrigel invasiveness was only evaluated in assays that rendered values in the corresponding control of cell migration exceeding 2,000 cells per square centimeter of membrane.

## Results and Discussion

**Figure 1:**
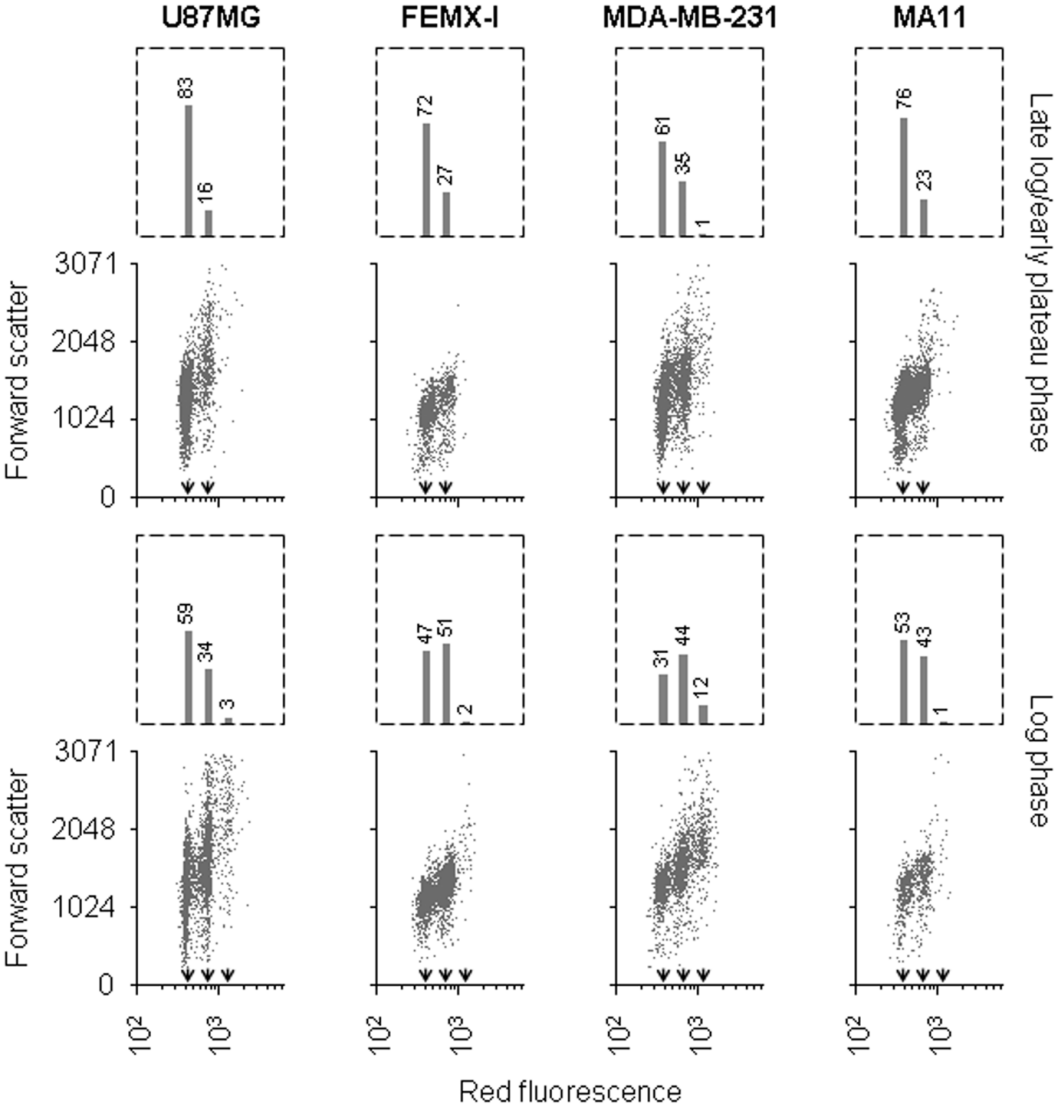
Detection in human tumor cell lines of cells containing four-fold DNA amount. Flow cytometric analysis of cultures of the tumor lines U87MG, FEMX-I, MDA-MB-231, and MA11, at either low (top panels) or high (bottom panels) rate of proliferation, showing the distribution of cells according to DNA content, expressed as intensity of red fluorescence, and forward scatter (arbitrary units); the bar graph on top of each panel shows the percentage of cells in the G0/G1 peak (lowest DNA amount per cell), and that of cells with 2- (including cells in S phase), and 4-fold the intensity of the cells of the G0/G1 peak. The average fluorescence of each of the three populations is indicated by arrowheads. In all the cell lines, a greater number of cells with four-fold DNA quantity is present in the rapidly proliferating culture, representing 3%, 2%, 12%, and 1% of the total cells for U87MG, FEMX-I, MDA-MB-231, and MA11, respectively.

Analyses of cell distribution based on DNA content allowed us to distinguish, in glioma cells, a separate peak containing cells with tumor-genome duplication in the G2/M phase of the cell cycle [14]. In analyses carried out on cell lines of melanoma, breast cancer, and glioblastoma, a subset of cells carrying four-fold the DNA quantity of the cells of the G0/G1 peak was noticeable in cultures harvested in the phase of exponential growth (Fig. 1). Instead, the subset of cells with four-fold amount of DNA was barely detectable in cultures that approached saturation density, with the exception of the breast carcinoma cell line MDA-MB-231, where it was diminished but still apparent. Hence, the populations of cells carrying 4-fold DNA quantity consisted of G2/M cells with twice the genome content of the main population, since their number of cells decreased as the rate of cell proliferation slowed down due to culture overgrowth. Therefore, given that cell fusion in cultures of tumor cells generates fully viable cells which keep an unchanged proliferation rate [14], [17], we assumed that the cells with 4-fold DNA amount accounted for cells that had undergone whole tumor-genome duplication. Among the four tumor cell lines analysed in log phase cultures, the largest cell population carrying duplicated tumor genome was detected in MDA-MB-231, and the smallest in MA11, following the sequence MDA-MB-231>U87MG>FEMX-I>MA11.

**Figure 2:**
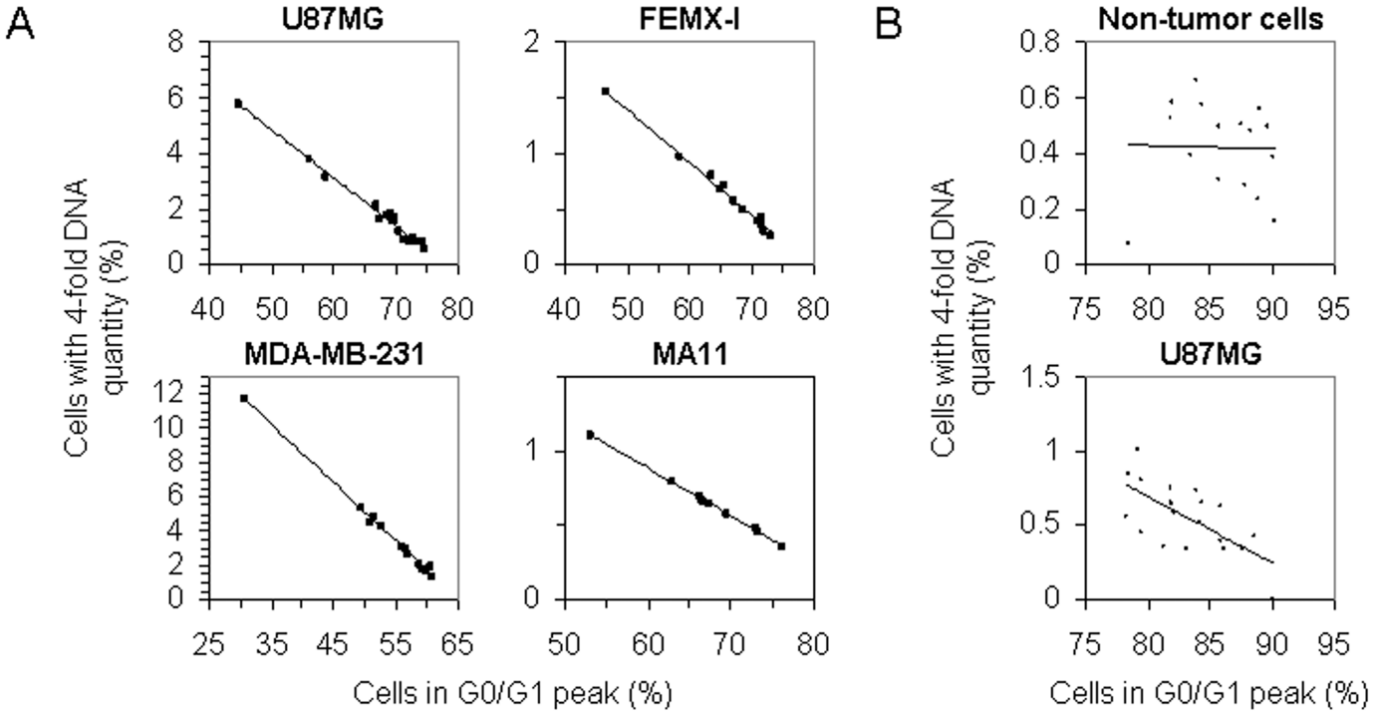
Relationship between cell proliferation in culture and number of tumor cells containing four-fold DNA amount. (A) Inverse relationship (r^2^>0.95) in U87MG, FEMX-I, MDA-MB-231, and MA11 cell lines, between the number of cells in the G0/G1 peak and that of cells carrying four-fold DNA amount, as quantitated in cultures of each cell line by flow cytometric analysis for content of DNA; both cell populations are expressed as fractions over the total number of cells analysed from each culture. (B) Lack of correlation in cultures of fibroblasts (top panel), *vs.* negative correlation in cultures of U87MG glioma cells (bottom panel), between number of cells in the G0/G1 peak and number of cells carrying four-fold DNA amount.

To assess the difference between the cell lines in their proportion of cells undergoing tumor-genome duplication, a number of cultures of each cell line were analyzed to quantitate the cell population displaying four-fold DNA amount in relation to the proliferation ratio. In each of the four tumor cell lines, there was an inverse proportionality between the number of cells in the G0/G1 peak and that of cells carrying four-fold amount of DNA (Fig. 2A); by contrast, in primary human fibroblasts, no apparent relation was found (Fig. 2B). The linear dependence of the proportion of cells with four-fold amount of DNA on the proliferation rate of the culture, which indicated that they were cells in the G2/M phase of the cell cycle, allowed to compare the cell lines in regard to their specific quantity of cells with duplicated tumor genome. The gradients of the fitting lines yielded ratios, relative to MA11, of 1-, 5-, and 11-fold, for FEMX-I, U87MG, and MDA-MB-231, respectively.

**Figure 3:**
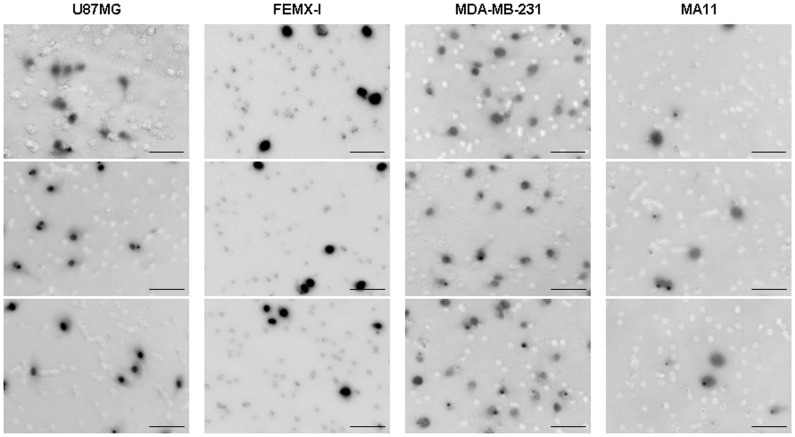
Matrigel invasion by U87MG, FEMX-I, MDA-MB-231, and MA11 tumor cell lines. Fluorescence microphotographs of representative areas of the underside of matrigel-coated membranes in transwell assays, showing that U87MG and MDA-MB-231 invade more efficiently through the matrigel, roughly by 4- and 7-fold, respectively, than MA11 and FEMX-I. The cells were fluorescently labeled with 4′,6-diamidino-2-phenylindole, appearing dark in the inverted images. Bars, 100 µm.

Having established the extent of tumor-genome duplication in each cell line, we then investigated the capacity of the cell lines to invade by proteolytic degradation. The invasiveness of the tumor cell lines was evaluated by using a chemotactic gradient to stimulate infiltration into a layer of matrigel matrix containing the basement membrane components heparan sulfate proteoglycan, laminin, collagen type IV, and entactin, and low levels of growth factors. The invasiveness varied from one cell line to another (Fig. 3); counting of the cells that reached the opposite side of the matrix layer yielded 1,278 (±410), 450 (±80), 2,415 (±671), and 337 (±135) cells/cm^2^ (mean±SD from 5–9 experimental replicates), for U87MG, FEMX-I, MDA-MB-231, and MA11, respectively.

**Figure 4:**
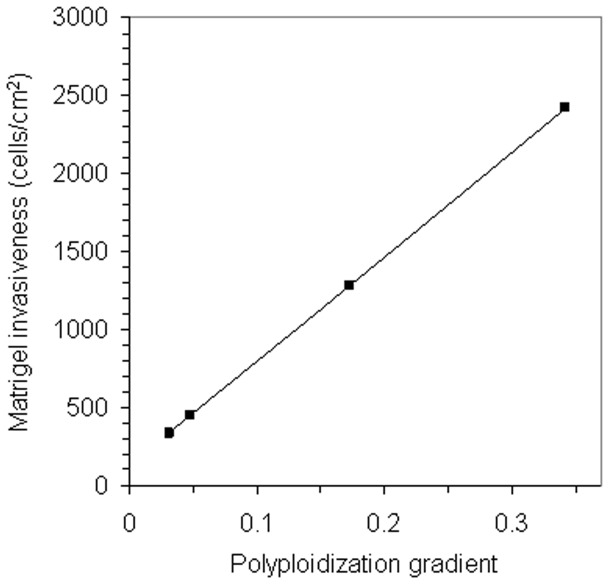
Relationship between invasiveness and polyploidization in tumor cells. Graph displaying a proportional ratio (r^2^>0.95) between the level of polyploidization in the tumor cell lines U87MG, FEMX-I, MDA-MB-231, and MA11, as expressed by the value of the gradient of the regression line, and average density of tumor cell invasion through matrigel.

The gradients of the fitted regression lines, expressing the extent of tumor-genome duplication in the cell lines, were then plotted against the densities of tumor cell invasion obtained in the matrigel assay. This revealed a direct proportionality between both values (Fig. 4), supporting tumor cell invasiveness as a determinant of tumor-genome duplication.

Tumor cell strategies other than pericellular proteolysis play a role in the invasive behaviour [18]. To pinpoint the contribution of protease activity to polyploidization, BB-2516, a broad-spectrum inhibitor of matrix metalloproteinases, was administered to U87MG cells as a single dose at 25 µM. Treatment for 4 days decreased the number of cells with tumor-genome duplication to 59%±2 of control (mean±SD from 2 experimental replicates). In addition, since critical metalloproteinases act in coordination with the CD44 membrane receptor [19], [20], we targeted CD44 with a specific antibody administered to tumor cells as four single daily doses at 0.1 nM. The antibody, while having no effect on the rate of cell proliferation, decreased the amount of cells with duplicated tumor genome, respect to cultures with isotype control, to 50%±19 (SD) of control in FEMX-I (P<0.05 by Student’s t-test; n = 5), and 59%±6 (SD) of control in MDA-MB-231 (P<0.01; n = 5). Altogether, the reduction by nearly one-half of the number of cells with tumor-genome duplication upon blockage of CD44 or matrix metalloproteinases, indicated that polyploidization was dependent on proteolytic activity.

In conclusion, we have shown, in a set of four human cancer cell lines, a direct relation between invasiveness and tumor-genome duplication. Taken together, our results suggest that cell fusion is a major source of polyploidization in tumor cells, and that it either adds to the polyploidization caused by other alterations, or is its primary cause. Furthermore, our data suggest that the acquisition by preneoplastic cells of mechanisms inducing invasion between daughter cells could contribute to the initiation of neoplastic growth.
